# The association between attachment insecurity and negative symptoms in clinical and non‐clinical populations: A systematic review and meta‐analysis

**DOI:** 10.1111/papt.70054

**Published:** 2026-03-26

**Authors:** Aiden Duffy, David Carmichael, Zsofia K. Takacs, Regina Murphy, Sean Harper, Tia McEwan, Karen Goodall

**Affiliations:** ^1^ University of Edinburgh Edinburgh UK; ^2^ NHS Lothian Edinburgh UK; ^3^ University of Glasgow Glasgow UK; ^4^ NHS Grampian Aberdeen UK

**Keywords:** attachment, meta‐analysis, negative symptoms, psychosis, systematic review

## Abstract

**Purpose:**

Attachment insecurity has been theorised to be associated with the development and maintenance of negative symptoms. Previous reviews examining the relationship between attachment insecurity and negative symptoms of psychosis have produced conflicting findings. Using meta‐analytic techniques, the current study sought to systematically appraise the relationship between attachment insecurity dimensions, total negative symptoms and domains of negative symptoms.

**Methods:**

A systematic review of literature published on electronic databases (PubMed, Ovid MEDLINE, EMBASE, PsycINFO) up to October 2025.

**Results:**

Twenty‐two studies met inclusion criteria (*n* = 6507; mean age 27.76 years; 35.1% male) and comprised clinical samples (*k* = 14) and non‐clinical samples (*k* = 9). Five studies achieved a low risk of bias score. A small, significant average association was found between negative symptoms and attachment anxiety in clinical and non‐clinical samples. A small, significant average association was found between attachment avoidance and negative symptoms in clinical samples, while a moderate‐sized, significant average association was found in non‐clinical samples. Differing strengths of association were found between attachment dimensions and each of the negative symptom domains.

**Conclusion:**

Future research should use measures that adhere to the NIMH consensus definition and should also explore the impact of attachment insecurity on individual negative symptoms.

**PRISMA/PROSPERO:**

This review was conducted in line with PRISMA guidance. The review protocol was registered on Prospero on the 16 August 2022. https://www.crd.york.ac.uk/PROSPERO/view/CRD42022353927.

## BACKGROUND

Negative symptoms, a core feature of psychotic disorders, are associated with decreased functioning and increased disability (Strauss & Cohen, 2017). Negative symptoms represent impaired function in areas such as motivation, pleasure, and expressivity (Correll & Schooler, 2020). While earlier studies employed differing definitions of negative symptoms, in 2006 a consensus statement developed by the NIMH agreed that negative symptoms comprise anhedonia (a decreased ability to derive enjoyment from activities), asociality (having little interest in a social life), avolition (a lack of the wilful initiation of activity), blunted affect (a decrease in the outward expression of emotion), and alogia (poverty of speech) (Kirkpatrick et al., 2006). Mild and transient negative symptoms, described as negative schizotypy, have been reported to occur in non‐clinical populations (Kwapil & Barrantes‐Vidal, 2015), differing from those experienced by people with psychosis in only their intensity, frequency, and the level of distress they cause (Johns & van Os, 2001).

Establishing the prevalence and correlates of negative symptoms across the psychosis continuum is complex. Assessment measures vary in their definition of negative symptoms, their recommended frequency of use, and their method of administration (Lincoln et al., 2017; Lyne et al., 2018). Measures developed prior to the NIMH consensus statement are affected by conceptual confusion and contain items unrelated to the construct, such as cognitive symptoms (Chang et al., 2021; Lincoln et al., 2017). In addition, they frequently utilize total scores when 1‐ and 2‐factor models of negative symptoms offer suboptimal fit, compared with 5‐factor models (Chang et al., 2021). A further confounding factor is that negative symptoms can be a primary manifestation of the underlying pathophysiology of psychosis, or a secondary cluster of symptoms that occur in response to factors such as anti‐psychotic‐related sedation, extrapyramidal side effects, comorbidities, or environmental effects (Mosolov & Yaltonskaya, 2022). For example, qualitative studies suggest that withdrawal from social interactions can arise from shame about mental health stigma or weight gain from medication (Moernaut & Vanheule, 2020).

Regardless of the cause, negative symptoms are associated with poor global functioning (Austin et al., 2015). People with lived experience of negative symptoms have described their relentless nature and the disabling impact that these symptoms have on their lives (Butcher et al., 2020). Despite their prognostic importance, currently available psychosocial and pharmacological treatments provide, at best, a modest improvement in symptoms (Lutgens et al., 2017). Seeking to refine psychosocial treatments, researchers are increasingly considering the role of attachment theory in the development and maintenance of negative symptoms (Griffiths & McLeod, 2019).

### Attachment theory

Attachment theory proposes the existence of an innate psychobiological system that organises the behaviour of humans at times of distress (Bowlby, 1969). Internal Working Models (IWMs), cognitive‐affective structures shaped by past experience of relationships, act as a mental representation of the self in relation to others to guide the person's care‐seeking strategies during times of distress (Mikulincer & Shaver, 2007). Individuals whose IWM confers a sense of relational security, a trust in others, and an expectation that they can cope with reasonable threats are described as having a secure attachment style (Bretherton et al., 1997). Those whose IWM predicts that others are inconsistent, unresponsive, or insensitive are described as experiencing attachment insecurity.

In adulthood, attachment is commonly conceptualised as two orthogonal dimensions of attachment anxiety and attachment avoidance (Brennan et al., 1998). Those high in attachment anxiety are described as having a strong desire to seek closeness when distressed and a tendency towards the intensification of their emotions as a care‐seeking strategy (Mikulincer & Shaver, 2019). Those high in attachment avoidance inhibit their emotional response to distress and hold a strong desire to minimise their dependence on others for support (Mikulincer & Shaver, 2019). Where the person's early experience of receiving care has been chaotic or threatening, individuals may exhibit both avoidant and anxious strategies when distressed. This type of attachment behaviour is described as disorganised (Main & Solomon, 1986). Insecure attachment style is strikingly over‐represented in people with psychosis (Carr et al., 2018) and has been associated with higher schizotypy in non‐clinical samples (Meins et al., 2008). This association suggests that the increased stress‐vulnerability and maladaptive coping associated with insecure attachment contribute to psychosis‐proneness (Barker et al., 2015). While associations between attachment insecurity and positive symptoms such as paranoia and voice‐hearing are well researched, the association between attachment insecurity and negative symptoms is less clear (Van Bussel et al., 2021).

The value of examining negative symptoms from an attachment perspective is supported by first‐person perspectives. A qualitative systematic review by Moernaut and Vanheule (2020) identified failing social interactions as a dominant theme. Participants with lived experience reported avoiding social contact through difficulties in expressing experiences, relating to others, and a feeling of detachment from the self. Butcher et al. (2020) also highlighted that negative symptoms entail substantial social disconnection through a preference for isolation when negative symptoms are present.

### Attachment and negative symptoms

Theoretically, attachment insecurity, particularly attachment avoidance, has been linked to the development of negative symptoms via IWMs (Berry et al., 2008). Attachment avoidance is associated with difficulties with closeness and a need for self‐reliance (Mikulincer & Shaver, 2019). At times of distress, for example, when faced with the anomalous internal experiences that characterise psychosis, there can be a propensity towards social withdrawal, overregulation of affect, and impaired mentalisation (Griffiths & McLeod, 2019). These factors have been theorised to mediate the relationship between attachment avoidance behaviour and negative symptoms (Berry et al., 2008; Griffiths & McLeod, 2019; Gumley, Schwannauer, et al., 2014; Harder, 2014). Negative symptoms can thus be viewed as a manifestation of a learned self‐regulatory response that developed to help the individual avoid negative affect in the face of challenging life events, for example, childhood maltreatment, earlier in the lifespan (Griffiths & McLeod, 2019). Indeed, exposure to childhood maltreatment is a risk factor for both attachment avoidance and psychosis (Baer & Martinez, 2006; Varese et al., 2012). Although this theory finds support from individual studies, systematic reviews exploring the association between attachment insecurity and negative symptoms are conflictual in their findings (Carr et al., 2018).

Modest associations between attachment avoidance and negative symptoms have been found in systematic reviews of clinical populations, while no associations were found between attachment anxiety and negative symptoms (Gumley, Taylor, et al., 2014; Korver‐Nieberg et al., 2014). In non‐clinical samples, evidence was limited to a systematic review conducted by Korver‐Nieberg and colleagues that reported modest positive associations between attachment avoidance and negative symptomatology in both clinical and non‐clinical populations. By contrast, a meta‐analysis conducted by Carr et al. (2018) concluded that, in non‐clinical samples, there was a moderate association between attachment avoidance and negative symptoms and a small association between attachment anxiety and negative symptoms. These associations were not replicated in clinical samples.

Since the meta‐analysis of Carr et al. (2018), (whose literature search was completed in 2015), no updated meta‐analyses have been conducted, despite increasing acceptance of attachment theory in the recovery from negative symptoms (Van Bussel et al., 2021). Furthermore, previous reviews of the relationship between attachment dimensions and negative symptoms, such as Carr et al. (2018), have tended towards defining negative symptoms as a homogenous concept, despite the use of divergent measures and the known pitfalls of conflating the five domains (Chang et al., 2021). Indeed, emerging evidence has demonstrated that each of the five domains of NS may have different etiological mechanisms and psychobiological correlates (Kaiser et al., 2017). Adopting a symptom‐specific approach, therefore, offers greater potential to identify conceptual overlap in measures, which would otherwise be obscured by total scores.

### Aims of this review

This study sought to clarify the relationship between attachment insecurity and negative symptoms, as conceptualised by the NIMH (Kirkpatrick et al., 2006). Specifically, it sought to clarify relationships between attachment insecurity dimensions (avoidance and anxiety) and total and specific negative symptoms in both clinical and non‐clinical populations, integrating relevant studies from January 1980 to October 2025.

## METHODS

### Systematic search

The review protocol was registered on PROSPERO (reg no. CRD42022353927). No ethical approval was sought for this review as it was based exclusively on already published data. A systematic literature search was carried out using five scientific databases: (*PubMed, Ovid, MEDLINE, EMBASE*, and *PsychINFO*) with the following, and related, search terms “attachment” AND (“psychosis” OR “schizophrenia” OR “schizotypy”) AND (“negative symptom*” OR “alogia” OR “blunted affect” OR “anhedonia” OR “avolition” OR “apathy” OR “asociality” OR “deficit syndrome”). Searching was carried out in three phases, with a final search in October 2025, resulting in 770 hits. The final search was conducted by two reviewers (AD and TMc). After the removal of duplicates, 642 papers remained.

### Eligibility screening

**FIGURE 1:**
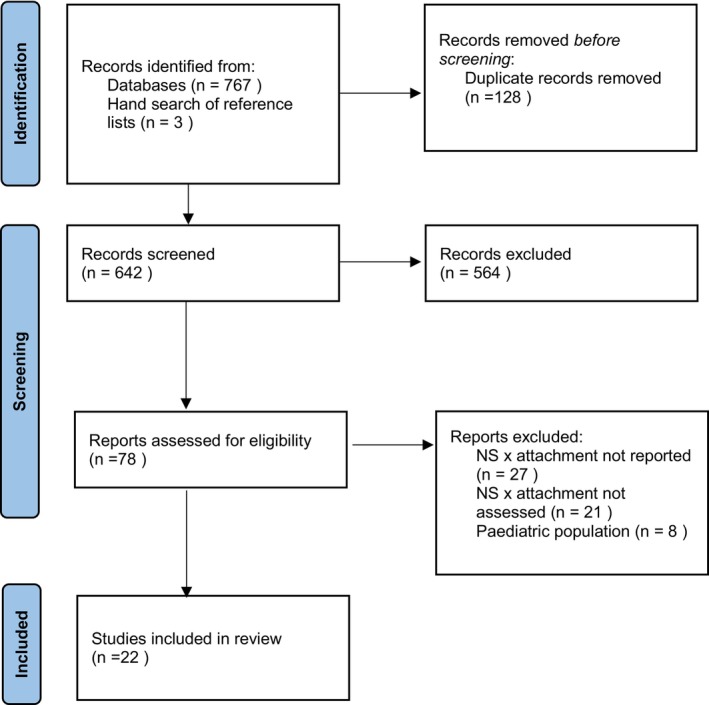
PRISMA flow chart depicting the search process.

The titles and abstracts of the 642 papers that were identified in the initial search were reviewed by AD and TMc, leading to 78 papers being retained. The full‐text of these papers were screened by the lead author and TMc with an almost perfect inter‐rater reliability prior to consensus discussion (Cohen's kappa = .82). A total of 56 papers were rejected due to (i) not reporting the association between negative symptoms and attachment, (ii) not assessing the relationship between these variables and (iii) including an unsuitable study sample (e.g., paediatric sample). Following this process, 22 studies remained for meta‐analysis (see Figure 1). This process was executed following PRISMA guidelines.

### Inclusion and exclusion criteria

Articles were included if they (1) used a validated measure of attachment, and a validated measure of negative symptoms (3) tested the association between attachment style and negative symptomatology (4) were an RCT, uncontrolled and/or non‐randomised study, cohort study, cross‐sectional study, or case–control study (5) were published in a peer‐reviewed journal between January 1980 and October 2025 (6) were in English.

Studies were excluded if they comprised: conference extract or poster, book chapter, theoretical or review articles, unpublished study, thesis, qualitative data, single case studies or dissertations.

### Coding process and type of data

A standardised coding sheet was used to extract data from the selected studies. Two reviewers (AD, TMc) completed the data extraction and coding process, with the senior researcher (KG) being consulted for guidance when case agreement could not be reached. There was almost perfect inter‐rater reliability prior to consensus discussion (Cohen's kappa = .96). The following information was extracted: (1) country of setting (2) clinical/non‐clinical setting (3) sample size (4) mean age of sample (5) gender of sample (6) attachment measure (7) negative symptomatology measure (8) correlation coefficients (between the numerical variables of attachment dimensions and negative symptoms, reported as *r*). For the analysis, correlation coefficients were coded as the effect size. Clinical samples were classified as those where participants were: (1) individuals diagnosed with schizophrenia spectrum disorders and (2) individuals experiencing first episode psychosis/other psychosis. Non‐clinical samples were classified as individuals from the general population.

### Quality assessment

Studies were assessed for risk of bias using an adapted version of the tool developed by Murphy et al. (2020). This tool was originally based on the AHRQ quality assessment tool (Williams et al., 2010), and assesses seven potential sources of bias: (1) sample selection, (2) sample size, (3) participant description, (4) validity of attachment measure, (5) validity of negative symptom measure, (6) handling of missing data and (7) data analysis. Each domain is given a score between 0 and 2, where 0 represents unclear or absent information, 1 represents partial information, and 2 represents that the information is clearly provided. A total score was calculated, then converted into a percentage representing how close the paper came to achieving its total maximum score, with higher scores indicating a lower risk of bias. In accordance with Murphy et al. (2020), a score of 80% was considered to indicate a low risk of bias. All papers were assessed and rated by AD. A random selection of 6 studies (26%) was independently assessed by RM. Quality ratings by the two independent reviewers (AD, RM) demonstrated a high level of inter‐rater reliability, with a Cohen's kappa of 0.96 prior to consensus discussion. Table 1 indicates that most studies did not achieve an 80% risk of bias score (*n* = 17).

**TABLE 1:** Summary of risk of bias assessments.

Study	Sample recruitment	Sample size	Sample description	Attachment assessment	Negative symptom assessment	Missing data	Adequate analysis	% of maximum total score
Akers et al. (2025)	Partial	Yes	Yes	Yes	Yes	Yes	Yes	93
Aydın et al. (2019)	No	No	Yes	Partial	Yes	No	Yes	50
Berry, Barrowclough, and Wearden (2006)	No	Partial	Yes	Yes	Yes	No	Yes	64
Berry, Barrowclough, and Wearden (2007)	No	Partial	Partial	Yes	Yes	No	Yes	57
Berry et al. (2008)	Yes	Partial	Yes	Yes	Yes	Partial	Yes	86
Burhan et al. (2022)	Yes	Yes	Yes	Yes	Yes	No	Yes	86
Chatziioannidis et al. (2019)	No	Partial	Yes	Yes	Yes	No	Yes	64
Degnan et al. (2022)	Partial	Partial	Yes	Yes	Yes	Partial	Yes	64
Farias et al. (2013)	No	Partial	Yes	Yes	Yes	No	Yes	64
França et al. (2020)	Yes	Partial	Yes	Yes	Yes	No	Yes	79
Grady et al. (2025)	Yes	Yes	Yes	Yes	Yes	Yes	Yes	100
Korver‐Nieberg et al. (2015)	Partial	Partial	Yes	Yes	Yes	No	Partial	64
Kvrgic et al. (2011)	Yes	Partial	Yes	Yes	Yes	No	Partial	71
Meins et al. (2008)	No	Partial	Yes	Yes	Yes	No	Yes	64
Ozdemir et al. (2024)	No	Yes	Yes	Yes	Yes	No	Yes	71
Ponizovsky et al. (2014)	Yes	Partial	Yes	Yes	Yes	No	Partial	71
Ringer et al. (2014)	Yes	Yes	Yes	Yes	Yes	No	Yes	86
Sheinbaum et al. (2013)	No	Partial	Partial	Yes	Yes	No	Partial	50
Sheinbaum et al. (2015)	No	Partial	Partial	Yes	Yes	No	Partial	50
Tiliopoulos and Goodall (2009)	No	Partial	Yes	Yes	Yes	No	Partial	57
van Dam et al. (2014)	Yes	Partial	Partial	Yes	Yes	No	Yes	71
Varela et al. (2021)	No	Partial	Yes	Yes	Yes	No	Partial	57

### Data analysis

Data were analysed using the Comprehensive Meta‐Analysis Version 3.0 software (Borenstein et al., 2021). For the quantitative synthesis, we used random effects meta‐analyses, one for attachment anxiety and one for attachment avoidance. The effect size in the present study was the bivariate correlation coefficient for the relationship between negative symptoms and (i) attachment anxiety and (ii) attachment avoidance. Where a study reported results for different subgroups or on different subscales, all effect sizes were extracted. The software averages the effect sizes of different subgroups or subscales in a study before entering that study in the meta‐analytic calculations.

Outliers were assessed with the standardised residuals, with values exceeding ±3.29 considered as outliers. We assessed publication bias by inspecting the symmetry of the funnel plot and using Egger's test. The average correlation coefficients were estimated using the random effects model. Heterogeneity was assessed by the *Q*‐statistics and *I* ^2^.

We also calculated the average effect sizes separately for clinical and non‐clinical samples, and contrasted these in a subgroup analysis. Finally, we inspected the results according to the different attachment measures and the different negative symptom subscales when data were available.

## RESULTS

### Descriptive statistics

From 22 studies reporting correlation data, 26 independent samples were identified (Table 2). In clinical samples, 12 effect sizes were found for the correlation between attachment anxiety and total negative symptoms, and 16 effect sizes were found for the correlation between attachment avoidance and total negative symptoms. In non‐clinical samples, 5 effect sizes for the correlation between attachment anxiety and total negative schizotypy were found, and 6 effect sizes for the correlation between attachment avoidance and total negative schizotypy were found. Negative symptom domains were measured in non‐clinical samples only: 2 effect sizes for the correlation between both attachment dimensions and constricted affect were found, 2 effect sizes for the correlation with asociality were found and 4 effect sizes for the correlation with anhedonia were found.

**TABLE 2:** Summary of study data extraction.

Source (author, date, country)	*N*	Sample, mean age (SD)	Gender (% male)	Attachment measure	Negative symptom measure	Negative symptomatology (NS) assessed (mean (SD))	Relevant findings
**Clinical samples**							
Akers et al. (2025) UK	132	Self‐reported psychosis‐spectrum disorder NR (median = 35)	25.8	PAM‐R	CAPE	Total NS 32.73 (8.74)	Significant association (*p* < .01) between total NS frequency scores and attachment anxiety (0.54), attachment avoidance (0.44), and disorganised attachment (0.50)
Aydın et al. (2019) Bosnia & Herzegovina	34	Schizophrenia 30.05 (7.41)	64	ECR‐R	PANSS	Total NS n.r.	Significant association (*p* < .01) between total NS scores and attachment avoidance (0.479)
Berry et al. (2008) UK	96	Schizophrenia, schizoaffective or nonaffective psychosis 44 (12.8)	68	PAM	PANSS	Total NS n.r.	Significant association (*p* = .019) between total negative symptoms and attachment avoidance (0.24)
Burhan et al. (2022) Turkey	80	Schizophrenia 40.34 (8.57)	71.2	PAM, AAS	PANSS	Total NS 24.66 (6.02)	None
Chatziioannidis et al. (2019) Greece	63	Inpatients with psychosis 40.44 (10.003)	69.84	ECR‐R	PANSS	Total NS n.r.	None
Degnan et al. (2022) UK	242	Self‐reported psychosis 33.17 (13.06)	63.6	PAM‐R	SNS	Total NS 21.19 (8.27) Expressive 8.21 (4.05) Experiential 12.98 (13.00)	Significant association (*p* < .001) between attachment avoidance and total neg. symptoms (0.58), expressive symptoms (0.472), and experiential symptoms (0.522). Significant association (*p* < .001) between disorganised attachment and total neg. symptoms (0.555), expressive symptoms (0.384), and experiential symptoms (0.548)
França et al. (2020) Portugal	41	Schizophrenia 53 (9.6)	63.4	AAS‐R	PANSS	Total NS n.r.	None
Grady et al. (2025) Ireland	71	Psychosis‐spectrum disorder 43 (11.88)	59.2	PAM‐R	SANS	Total NS 5.76 (3.36) Expressive 1.97 (1.83) Experiential 3.79 (2.06)	Significant association (*p* < .01) between total NS and attachment avoidance (0.436) and disorganised attachment (0.294). Between expressive NS and attachment avoidance (0.313). Between experiential NS and attachment avoidance (0.424) and disorganised attachment (0.345)
Korver‐Nieberg et al. (2015) Netherlands, Israel, UK	500	Schizophrenia, schizoaffective or non‐affective psychosis 37.5 (11.7)	80.4	RQ	PANSS	Total NS 19.1 (7.9)	None
Kvrgic et al. (2011) Switzerland	127	Schizophrenia or schizoaffective disorder 44.6 (11.53)	66	PAM	PANSS	Total NS 14.44 (4.97)	None
Ponizovsky et al. (2014) Israel	101	Schizophrenia 37.5 (11.1)	90.1	RQ	PANSS	Total NS 20.0 (4.9)	Significant association (*p* < .05) between total NS scores and attachment avoidance (−0.2)
Ringer et al. (2014) USA	52	Schizophrenia 46.64 (9.15)	100	ECR	PANSS	Total NS 19.85 (5.22)	None
Varela et al. (2021) Chile	51	Non‐affective psychosis 35 (10)	82.4	PAM	PANSS	Total NS 15.04 (5.05)	None
**Mixed and non‐clinical samples**							
Berry, Band, et al. (2007) UK	304	Students 21 (nr)	22	PAM	O‐LIFE	Introvertive Anhedonia n.r.	Significant association (*p* < .01) between introvertive anhedonia and both attachment avoidance (0.50) and attachment anxiety (0.26)
Berry, Wearden, et al. (2006) UK	323	Students 21 (nr)	28	PAM, RQ	r‐SAS	Social Anhedonia n.r.	Significant association between social anhedonia and both attachment avoidance (*p* < .001, .44) and attachment anxiety (*p* = .004, .19). Only the avoidance and social anhedonia relationship remained significant when controlling for negative affect
Farias et al. (2013) UK	114 (spiritual believers), 86 (traditional believers)	Community 37.37 (13.46), 32.40 (15.70)	26.3, 25.6	ECR	O‐LIFE	Introvertive Anhedonia 3.00 (2.09)	Significant association (*p* < .001) between introvertive anhedonia and both attachment avoidance (0.46) and attachment anxiety (0.41) in the spiritual group. Significant association between introvertive anhedonia and both attachment avoidance (*p* < .001, .48) and attachment anxiety (*p* < .05, .34) in the religious group
Meins et al. (2008) UK	154	Students 20.6 (2.98)	43.5	RQ	SPQ	Constricted affect, no friends, social anxiety n.r.	Significant association (*p* < .004) between attachment avoidance and constricted affect (0.34), no friends (0.31), and social anxiety (0.26). Significant association (*p* < .004) between attachment anxiety and constricted affect (0.31), no friends (0.25), and social anxiety (0.33)
Ozdemir et al. (2024) UK	1263	Community 27.84 (4.77)	12	PAM‐R	MSS‐B	Total NS n.r.	Significant association (*p* < .0004, Bonferroni‐corrected) between total NS scores and attachment avoidance (0.54) and disorganised attachment (0.41)
Sheinbaum et al. (2013) Spain, USA	547 (Spain), 1425 (USA)	Students 20.6 (4.11) (Spain), 19.8 (3.93) (USA)	16.8 (Spain), 23.5 (USA)	RQ	WSS	Total NS Spanish −0.18 (0.86), USA 0.01 (1.0)	Spain: Significant association (*p* < .001) between total negative schizotypy and secure attachment (−0.21), dismissing attachment (0.22) and fearful attachment (0.25). USA: Significant association (*p* < .001) between total negative schizotypy and secure attachment (−0.34), dismissing attachment (0.28) and fearful attachment (0.28)
Sheinbaum et al. (2015) Spain	214	Students 21.4 (2.4)	22	ASI	CAARMS	Total sub‐clinical NS 1.51 (2.39)	Significant association (*p* < .05) between total sub‐clinical NS scores and angry‐dismissive attachment (0.17)
Tiliopoulos & Goodall, 2009 UK	161	Students and community 46.9 (18.9)	31.7	ECR	SPQ	Constricted affect, no friends, social anxiety 6.22 (5.76)	Significant association (*p* < .05) between attachment avoidance and constricted affect (0.35), social anxiety (0.25) and having no friends (0.39). Significant association (*p* < .05) between attachment anxiety and constricted affect (0.22) and social anxiety (0.31)
van Dam et al. (2014) Netherlands	131 (patients), 123 (siblings), 72 (community)	Psychotic disorder 31.19 (10.58), Siblings 30.89 (8.12), Community 30.89 (7.47)	84 (patients), 47.2 (siblings), 63.9 (community)	PAM	CAPE	Total NS n.r.	Patients: Significant association (*p* < .001) between total negative schizotypy and both attachment avoidance (0.27) and attachment anxiety (0.32). Siblings: Significant association (*p* < .001) between total negative schizotypy and both attachment avoidance (0.49) and attachment anxiety (0.57). Community: Significant association (*p* < .001) between total negative schizotypy and both attachment avoidance (0.41) and attachment anxiety (0.43)

### Samples

Thirteen studies were based solely on clinical samples comprising people diagnosed with a schizophrenia‐spectrum disorder. Eight studies were based on non‐clinical samples. One study (van Dam et al., 2014) included both a clinical and a non‐clinical sample (comprising patients' siblings and a community sample). The studies yielded a total of 6507 (M = 295.7, range 34–1972) participants with a mean age of 27.76 years. There were 2283 (35.1%) male participants across all studies. Male participants were a majority in the clinical studies (69.5%) but a minority in the non‐clinical studies (24.1%). A majority of the overall participants were from non‐clinical populations (*n* = 4917, 75.6%).

Attachment measures: Adult Attachment Scale (AAS: Collins & Read, 1990); Adult Attachment Scale Revised (AAS‐R: Collins, 1996); Experiences in Close Relationships (ECR: Brennan et al., 1998); Experiences in Close Relationships Revised (ECR‐R: Fraley et al., 2011); Psychosis Attachment Measure (PAM: Berry, Barrowclough, & Wearden, 2006); Psychosis Attachment Measure Revised (PAM‐R: Pollard et al., 2020); Relationship Questionnaire (RQ: Bartholomew & Horowitz, 1991). Negative symptom measures: Comprehensive Assessment of the At‐Risk Mental States (CAARMS: Yung, McGorry, et al., 2005); Community Assessment of Psychic Experience (CAPE: Stefanis, Avramopoulos, et al., 2002); Multidimensional Schizotypy Scale‐Brief (MSS‐B: Gross et al., 2018); Oxford‐Liverpool Inventory of Feelings and Experiences scale (O‐LIFE: Mason et al., 1995); Positive and Negative Syndrome Scale (PANSS: Kay et al., 1987); Self‐Evaluation of Negative Symptoms (SNS: Dollfus et al., 2016); Revised Social Anhedonia Scale (r‐SAS: Eckblad et al., 1982); Scale for the Assessment of Negative Symptoms (SANS: Andreasen, 1982); Schizotypal Personality Questionnaire (SPQ: Raine, 1991); Wisconsin Schizotypy Scales (WSS: Kwapil et al., 2008).

### Qualitative synthesis

#### Relationship between insecure attachment and negative symptoms in clinical samples

Fourteen studies investigated associations between insecure attachment dimensions and negative symptoms in clinical samples; seven found no association between attachment insecurity dimensions and total negative symptoms. All of the studies that reported no association used the PANSS to assess negative symptoms.

Two studies that used the PANSS reported positive associations between attachment avoidance and negative symptoms (*r* = .24, Berry et al., 2008; *r* = .48, Aydın et al., 2019). However, when the data from Berry et al. (2008) were combined with that of two other studies (Korver et al., 2012; Ponizovsky et al., 2007), the association between attachment avoidance and NS was no longer significant (Korver‐Nieberg et al., 2015).

van Dam et al. (2014) reported a positive association (*r =* .27) between attachment avoidance and negative symptoms as measured by the CAPE. The strongest significant positive association between attachment avoidance and total NS (*r* = .58) was reported by Degnan et al. (2022). This study was the only study to use the Self‐evaluation of Negative Symptoms scale, a measure that directly aligns with the five domains outlined in the NIMH consensus statement (SNS; Dollfus et al., 2016; Kirkpatrick et al., 2006). A further strength of this study was its use of the PAM‐R. This allowed the authors to report a significant association between disorganised attachment and total negative symptoms (*r* = .55). Mediation analysis found that the relationship between childhood trauma and total NS scores was fully mediated by disorganised attachment (β = .32) and dissociation (β = .16). Akers et al. (2025) and Grady et al. (2025) also made use of the PAM‐R and reported a similar strength of association between disorganised attachment and total negative symptoms scores (*r* = .50; *r* = .294). These studies used measures of negative symptoms that allowed an assessment of the relationship between disorganised attachment and both experiential and expressive negative symptoms. Experiential symptoms were significantly associated in both studies (*r* = .548, Akers et al., 2025; *r* = .345, Grady et al., 2025). Only Akers et al. (2025) found a significant association between disorganised attachment and expressive symptoms (*r* = .384).

A single study (Ponizovsky et al., 2014) reported a negative association between attachment avoidance, as measured by the Relationship Questionnaire (RQ) and negative symptoms (*r* = −.20).

In relation to attachment anxiety, two of the fourteen studies reported an association (*r* = .54; *r* = .32) with total negative symptom scores based on the CAPE frequency scores (Akers et al., 2025; van Dam et al., 2014).

#### Relationship between insecure attachment and negative symptoms in non‐clinical samples

Three studies, two of which included two non‐clinical samples within each study, found significant positive associations between attachment avoidance (or dismissing attachment) and total negative schizotypy scores (*r* = .22 to *r* = .54) (Ozdemir et al., 2024; Sheinbaum et al., 2013; van Dam et al., 2014). Sheinbaum et al. (2013) and Ozdemir et al. (2024) reported on the association between fearful or disorganised attachment (high attachment anxiety and high attachment avoidance) and schizotypy. Significant positive associations were reported in all three samples across these two studies (*r* = .25 to *r* = .41).

A follow‐up to the 2013 study was conducted by Sheinbaum et al. (2015). This study utilised the Attachment Style Interview (ASI: Bifulco et al., 2002) to classify participants into enmeshed, fearful, withdrawn and angry‐dismissive categories. Enmeshed and fearful categories represent an anxious profile, while withdrawn and angry‐dismissive categories represent an avoidant profile. This study supported the findings of Sheinbaum et al. (2013) in identifying a positive association between angry‐dismissive attachment and total negative schizotypy (*r* = .17). Similar to Sheinbaum et al. (2013), this further study scored poorly in the risk of bias assessment and had an overwhelmingly female sample (78%).

Only one study, van Dam et al. (2014), reported significant positive associations between attachment anxiety and total negative schizotypy (siblings of people with a psychotic disorder [*r* = .57]; healthy controls [*r* = .43]).

#### Relationship between insecure attachment and negative symptom domains in non‐clinical samples

A preponderance of studies examining associations between attachment dimensions/styles and negative symptoms relied on total scores. This is particularly the case with studies of clinical samples. A small number of non‐clinical studies have investigated potential associations between the five domains and dimensions of attachment insecurity.

Three studies reported positive associations between attachment avoidance and anhedonia, ranging from *r* = .44 to *r* = .50, and associations between attachment anxiety and anhedonia (*r* = .19 to *r* = .41).

Two of these studies provided additional statistical analysis. Berry, Wearden, et al. (2006) reported that their finding of a small positive association between attachment anxiety and social anhedonia (*r* = .19) was no longer significant when controlling for negative affect. However, the positive association (*r* = .44) between social anhedonia and attachment avoidance remained significant when controlling for negative affect. The second study, Berry, Barrowclough, and Wearden (2007), conducted a hierarchical multiple regression to establish if the attachment insecurity dimension would predict introvertive anhedonia independently of other early interpersonal experiences. When the effect of these experiences was controlled for, attachment anxiety did not explain significant variance in introvertive anhedonia.

A further two studies reported positive associations between attachment avoidance and asociality (No Close Friends sub‐scale of the SPQ) (*r* = .31 to *r* = .39). Only one of these studies (Meins et al., 2008) reported a significant positive association between attachment anxiety and the No Close Friends sub‐scale (*r* = .31). Similar to the other studies that measured non‐clinical negative samples, in these studies were predominantly female (56.5%–68.3%).

Two studies (Meins et al., 2008; Tiliopoulos & Goodall, 2009) assessed the relationship between attachment avoidance and blunted affect, reporting positive associations (*r* = .34 to *r* = .35). As outlined in Table 3, the interpersonal dimension of the SPQ is a poor fit for the five‐factor model of negative symptoms. An overview of the negative symptom measures used, and their adherence to the five‐factor model, can be found in Table 3.

**TABLE 3:** Summary of negative symptom measures.

Measure of negative symptoms	Description of measure	Negative symptom sub‐scales	Adherence to the 5‐factor model
Positive and Negative Symptoms Scale (PANSS) (Kay et al., 1987)	Clinician Clinician‐administered semi‐structured interview consisting of 30 items. Focuses on symptoms across the past week	7 items are combined to give a total negative symptoms score	Item N5, Difficulty in Abstract Thinking and N7 Stereotyped Thinking represent cognitive symptoms which do not fit with the now established 5‐factor model (Shafer & Dazzi, 2019)
Self‐evaluation of Negative Symptoms (SNS) (Dollfus et al., 2016)	A 20‐item self‐report measure. Patients complete 3‐point Likert scales to show their agreement with statements about their feelings over the past week	Subsets of four items are combined to create sub‐scores, which capture the 5 domains of symptoms	Good face and content validity; captures 5 domains of symptoms (Mach et al., 2015)
Scale for the Assessment of Negative Symptoms (SANS) (Andreasen, 1982)	A 25‐item clinician‐rated measure. Five sub‐scales capture affective blunting, alogia, avolition‐apathy, anhedonia‐asociality and attention	Individual domain scores can be utilised or combined into a composite score	Assesses all five factors; however, it also includes items that assess attention, which is not an aspect relevant to current conceptualisations of negative symptoms (Galderisi et al., 2021)
Community Assessment of Psychic Experiences (CAPE) (Stefanis, Hanssen, et al., 2002)	A 42‐item self‐report measure. Patients complete 4‐point Likert scales indicating the frequency and distress caused by psychosis spectrum experiences across the lifespan	14 items are combined to create a total negative symptoms score	Assesses for asociality, anhedonia and amotivation. However, no significant association between these three dimensions and scores on the PANSS (Lincoln et al., 2017)
Schizotypal Personality Questionnaire (SPQ) (Raine, 1991)	A 74‐item self‐report measure consisting of yes/no items	25 items across three sub‐scales (constricted affect, social anxiety, and no close friends) are combined to create an interpersonal dimension, which is proposed to align with negative schizotypy	Interpersonal dimension is a poor fit for the 5‐factor model of negative symptoms and shows stronger correlations with neuroticism (Gross et al., 2014)
Oxford Liverpool Inventory of Feelings and Experiences (O‐LIFE) (Mason et al., 1995)	A 104‐item self‐report measure consisting of yes/no items. Four sub‐scales capture Unusual perceptual experiences, Cognitive disorganisation, Introvertive Anhedonia, and Impulsive nonconformity	27 items are combined to create a total overall score for introvertive anhedonia	Scores for introvertive anhedonia do not show a significant relationship with scores on the SANS*; suggested that the scale may be measuring normal personality variation as opposed to pathology (Cochrane et al., 2010)
Revised Social Anhedonia Scale (r‐SAS) (Eckblad et al., 1982)	A 40‐item self‐report measure consisting of true/false items	All items are combined to provide a total overall score for social anhedonia	Assesses for social anhedonia conceptualised as: social indifference, asociality, lack of social enjoyment. Although designed as a measure of negative schizotypy, it has been found to tap both positive and negative symptom dimensions (Lewandowski et al., 2006)
The Comprehensive Assessment of At‐Risk Mental States (CAARMS) (Yung, Pan Yuen, et al., 2005)	The clinician administered a semi‐structured interview. Seven sub‐scales, each scored between 1 and 6, capture different aspects of psychosis onset. Designed to be used across time (e.g., monthly) to monitor psychosis onset in people with at‐risk mental states	Negative symptoms sub‐scales assess alogia, anhedonia, and avolition. Three sub‐scales are combined to provide a total negative symptoms score	Negative symptoms sub‐scale shows strong correlations with positive symptoms sub‐scale. Does not include blunted affect or asociality. Conceptualisation of alogia, anhedonia, and avolition does not provide a good fit with the 5‐factor domains (Strauss et al., 2020)
Multidimensional Schizotypy Scale‐Brief (MSS‐B) (Gross et al., 2018)	A 38‐item self‐report measure consisting of true/false items. Used to measure schizotypy—a personality construct related to schizophrenia‐spectrum disorders	13 items are combined to create a total negative schizotypy score	Full version of the MSS assesses for social disinterest, flat affect, anhedonia, alogia, anergia, and avolition. MSS‐B has comparable psychometric properties (Gross et al., 2018)
Wisconsin Schizotypy Scales (WSS)	A collection of four self‐report scales consisting of true/false items: Perceptual Aberration Scale, Magical Ideation Scale, Physical Anhedonia Scale, Revised Social Anhedonia Scale	See r‐SAS above	See r‐SAS above

Due to the limited number of studies reporting the association between insecure attachment and negative symptom domains, a sufficiently powered subgroup analysis could not be undertaken.

### Meta‐analysis of studies reporting on correlational analyses

#### Association between attachment anxiety and negative symptoms

**FIGURE 2:**
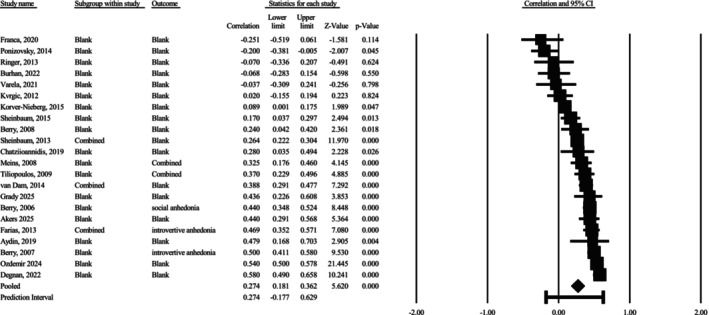
Forest plot of correlations between attachment avoidance and negative symptoms.

In total, 19 studies (4931 participants) reported the correlation between attachment anxiety and negative symptoms. As shown in Figure 2, correlation coefficients ranged between −0.134 and 0.540. There were no outlying effect sizes. The average correlation was small but significant (*r* = .193, 95% CI: 0.108, 0.276, *p* < .001). This was a highly heterogeneous effect (*Q* (18) = 130.51, *p* < .001, *I* ^2^ = 86.21). There were no signs of publication bias with a non‐significant Egger's test (intercept: 1.90, *p* = .10) and a symmetrical distribution on the funnel plot. The year of publication did not have an effect on the correlations (coefficient: .002, *p* = .86).

We then examined the average correlation coefficient for studies reporting on clinical and non‐clinical samples separately. For the 12 studies that reported on a clinical sample, we found a small but significant average correlation (*r* = .171, 95% CI: 0.045, 0.293, *p* = .008). This effect was heterogeneous (*Q* (11) = 24.17, *p* < .001, *I* ^2^ = 79.69). It should be noted that the year of publication seemed to be related to the correlations when inspecting the scatterplot. More specifically, more recent studies seem to report somewhat larger correlations. This effect did not reach significance (coefficient: .01, *p* = .44); however, statistical power was likely very low in this analysis. Similarly, synthesising the results of 8 studies that reported on a nonclinical sample, there was a small but significant average correlation (*r* = .243, 95% CI: 0.115, 0.363, *p* < .001), which was highly heterogeneous (*Q* (7) = 81.01, *p* < .001, *I* ^2^ = 91.36). There was no effect of year of publication on the correlations based on a nonsignificant effect (coefficient: .004, *p* = .85) and inspection of the scatterplot.

When conducting a subgroup analysis to contrast these two average effect sizes, we had to exclude one study that reported on both clinical and nonclinical subgroups. The subgroup analysis showed no significant difference between the 11 studies with a clinical sample (*r* = .156, 95% CI: 0.020, 0.285, *p* = .02) and the 7 studies with a nonclinical sample (*r* = .197, 95% CI: 0.090, 0.300, *p* < .001, *Q* (1) = 0.228, *p* = .63). Results according to the different attachment measures and the different negative symptom subscales are reported in Table 4. Thus, it can be concluded that there is a small but significant relationship between attachment anxiety and negative symptoms in both clinical and non‐clinical samples.

**TABLE 4:** Results according to measures of attachment and negative symptoms.

		Anxious attachment	Avoidant attachment
Attachment measure	AAS‐R	1 study *r* = −.134, 95% CI: −0.424, 0.181, *p* = .41	1 study *r* = −.251, 95% CI: −0.519, 0.061, *p* = .11
	ASI	1 study *r* = .080, 95% CI: −0.055, 0.212, *p* = .24	1 study *r* = .170, 95% CI: 0.037, 0.297, *p* = .01
	ECR	5 studies *r* = .222, 95% CI: 0.074, 0.360, *p* = .004	5 studies *r* = .326, 95% CI: 0.155, 0.478, *p* < .001
	PAM	8 studies *r* = .273, 95% CI: 0.141, 0.395, *p* < .001	10 studies *r* = .345, 95% CI: 0.215, 0.462, *p* < .001
	RQ	4 studies *r* = .082, 95% CI: −0.011, 0.173, *p* = .09	4 studies *r* = .137, 95% CI: −0.031, 0.297, *p* = .11
Negative symptom subscales	Anhedonia subscales	3 studies *r* = .272, 95% CI: 0.165, 0.373, *p* < .001	3 studies *r* = .469, 95% CI: 0.414, 0.521, *p* < .001
	Constricted affect	2 studies *r* = .265, 95% CI: 0.158, 0.365, *p* < .001	2 studies *r* = .345, 95% CI: 0.243, 0.439, *p* < .001
	No friends	2 studies *r* = .190, 95% CI: 0.070, 0.304, *p* = .002	2 studies *r* = .352, 95% CI: 0.250, 0.445, *p* < .001

Abbreviations: ASI, Attachment Style Interview; CI, Confidence interval; ECR, Experiences in Close Relationships; PAM, Psychosis Attachment Measure; RQ, Relationship Questionnaire.

#### Association between attachment avoidance and negative symptoms

Twenty‐two studies (6507 participants) reported the correlation between attachment avoidance and total negative symptoms. As shown in Figure 2, correlation coefficients ranged between −0.251 and 0.580. There were no outlying effect sizes. The average correlation was small but significant (*r* = .274, 95% CI: 0.181, 0.362, *p* < .001). This was a highly heterogeneous effect (*Q* (21) = 272.19, *p* < .001, *I* ^2^ = 92.29). There were no signs of publication bias with a non‐significant Egger's test (intercept = −1.81, *p* = .21) and a symmetrical spread on the funnel plot. Year of publication did not have a significant effect on the correlations in a meta‐regression analysis (coefficient: .001, *p* = .89).

When inspecting the average effect sizes of the 14 studies that included a clinical sample, a small but significant average correlation (*r* = .175, 95% CI: 0.025, 0.318, *p* = .02) was found. This was a heterogeneous effect (*Q* (13) = 115.77, *p* < .001, *I* ^2^ = 88.77). Based on the inspection of the scatterplot, more recent studies seemed to report larger correlations. This effect did not reach significance (coefficient: .02, *p* = .13), however, statistical power was likely very low and this nonsignificant result should be interpreted cautiously. In contrast, non‐clinical samples showed a moderate‐sized, significant average correlation (*r* = .400, 95% CI: 0.297, 0.494, *p* < .001), which was heterogeneous (*Q* (8) = 109.37, *p* < .001, *I* ^2^ = 92.69). There was no effect of the year of publication on the correlations based on a nonsignificant regression (coefficient: .005, *p* = .68) and inspection of the scatterplot. In order to statistically contrast the two, we conducted a subgroup analysis, in which we had to exclude the study that included clinical and nonclinical subsamples. There was a significant difference between the 13 studies with a clinical sample (*r* = .167, 95% CI: 0.003, 0.322, *p* = .046) and the 8 studies with a nonclinical sample (*r* = .392, 95% CI: 0.279, 0.495, *p* = .031, *Q* (1) = 5.324, *p* = .021). This sub‐group analysis suggests there was a moderate significant correlation between attachment avoidance and negative symptoms in non‐clinical samples, while only a small but significant association in clinical samples.

## DISCUSSION

### Relationships between attachment dimensions and total negative symptoms

The current meta‐analysis draws on previous reviews to examine relationships between attachment insecurity dimensions and negative symptoms in clinical and non‐clinical samples, including an additional ten studies since the meta‐analysis conducted in 2018 by Carr and colleagues. Based on cross‐sectional correlation results, small but significant correlations were found for the relationship between attachment anxiety and total negative symptoms in both clinical and nonclinical samples, with no significant differences between the clinical and non‐clinical groups. There was a moderate significant average correlation coefficient for the association between attachment avoidance and total negative symptoms in non‐clinical samples, and a small but significant average association in the clinical samples. The difference between the clinical and non‐clinical groups was significant.

The emergence of significant associations in clinical samples may reflect improved measurement precision. Earlier reliance on the PANSS may have obscured associations due to poor construct validity, whereas several more recent studies (Akers et al., 2025; Degnan et al., 2022; Grady et al., 2025) employed measures more closely aligned with the NIMH conceptualisation of negative symptoms. These studies also used psychosis‐specific attachment measures, which may have enhanced sensitivity to associations. However, meta‐regression indicated that publication year did not significantly moderate effect size, suggesting that differences in construct alignment and measurement approach are more relevant than recency. It remains unclear whether the shift away from PANSS reflects greater recognition of the NIMH definition of negative symptoms or is related to the well‐recognised challenges associated with its administration in clinical settings (Østergaard et al., 2017).

In contrast to the previous meta‐analysis conducted by Carr et al. (2018), the inclusion of additional contemporary studies presents a pattern of results broadly aligned with the continuum model of psychosis, which suggests that subclinical negative symptoms are associated with the same attachment‐related risk factors that contribute to psychosis, including deficits in mentalising, relationship difficulties, and negative self‐schemas (Butter et al., 2023; Cowan et al., 2021; De Salve et al., 2023).

According to the continuum model, there is an expectation that we would see attenuated relationships between concepts in non‐clinical groups compared to clinical groups. However, cross‐sectional studies cannot address the potentially bidirectional effect of attachment orientation and negative symptoms. Here, attachment avoidance is of specific interest. Attachment avoidance has been linked to low mentalising, deactivation of positive affect, and a lack of trust in relationships, implicating it in the unfolding of negative symptoms (Gumley et al., 2018). In turn, negative symptoms amplify the desire for isolation and difficulties in relating to others (Butcher et al., 2020; Moernaut & Vanheule, 2020) and reductions in trust that are specific to psychosis experience (Castien, 2017). Thus, attachment insecurity and negative symptoms have the potential to have a mutually reinforcing relationship over time, particularly in clinical samples. Future studies could consider the use of cross‐lagged longitudinal designs to assess these issues.

Four studies included a measure of disorganised attachment. The associations found in these studies indicate that disorganised attachment may play a role in the development and maintenance of negative symptoms. Disorganised attachment emerges from early caregiving environments that are characterised by significant adversity or threat, such as neglect or physical abuse. People who have grown up in such adverse environments may have an internal working model that drives uncoherent and contradictory behaviour at times of distress (Pollard et al., 2023). Individuals with a disorganised attachment have been found to experience greater clinical impairment and a higher level of positive symptoms when experiencing psychosis (Bucci et al., 2017).

Degnan et al. (2022) provide evidence to suggest that disorganised attachment may serve as a mediating mechanism in the pathway between childhood trauma and negative symptoms. Theoretically, the impact of living in a traumatic environment as a child, and the relational and affective difficulties that emerge from this experience, are suggested to crystallise into long‐term impairments in motivation, emotional expression, and other domains of negative symptoms, in a way that may not occur in non‐traumatised individuals with schizophrenia (Pruessner et al., 2021). Furthermore, disorganised attachment is associated with deficits in mentalising, a capacity that underpins self‐agency, social behaviour, and other psychological processes that are impaired in people with negative symptoms (Lysaker et al., 2021). Overall, these findings suggest that interventions targeting attachment insecurity, particularly when rooted in childhood trauma, may benefit individuals with negative symptoms (Humphrey et al., 2022).

### Relationships between attachment dimensions and negative symptom domains

At the outset of this study, there had been an aim to further interrogate relationships between attachment insecurity and domains of negative symptoms, in line with symptom‐specific approaches. However, correlation coefficients were available for non‐clinical samples only in five studies. As such, sub‐group analyses could not be completed due to a lack of statistical power. The extent to which specific domains of negative schizotypy are associated in clinical samples also remains unknown due to a reliance on total scores of negative symptoms in all clinical studies. Further studies, including measures of the domains of negative symptoms, rather than aggregate scores, are warranted.

### Limitations

The heterogeneity of the measures used and differences in how the measures operationalised the constructs under investigation must be acknowledged. Despite the NIMH consensus statement being over fifteen years old (Kirkpatrick et al., 2006), researchers continue to use measures of negative symptoms that do not align with the five‐factor model. The majority of studies (*n* = 10) on clinical samples use the negative symptom subscale of the Positive and Negative Syndrome Scale (PANSS). Subsequent principal components analyses have indicated that some negative symptoms do not load onto the same factor; therefore, captured variance is reduced (van der Gaag, Cuijpers, et al., 2006; van der Gaag, Hoffman, et al., 2006). Furthermore, the total negative symptom scores on the PANSS include items, such as difficulty in abstract thinking and stereotyped thinking, which do not feature in current conceptualisations of negative symptoms (Kirkpatrick et al., 2006). Complicating this issue, some authors have suggested that the five‐factor model itself is incomplete and overlooks the domain of emotional experience (e.g., emotional breadth, depth and complexity) (Alphs, 2006).

The validity of attachment measures when used with people experiencing psychosis must also be considered when examining the results of the clinical studies. People experiencing psychosis are more likely to be socially isolated (Giacco et al., 2016). This can make it difficult for them to accurately complete relationship‐specific measures of attachment such as the ECR (Berry, Barrowclough, & Wearden, 2006). The PAM was designed to overcome the issues that were faced by people with psychosis when using attachment measures (Berry, Wearden, et al., 2006). However, self‐report measures of attachment could underestimate attachment, especially in people with attachment avoidance (Gumley, Schwannauer, et al., 2014).

Throughout the studies, measures of both negative symptoms and attachment were administered at a single time point. Ratings of negative symptoms can change as people with psychosis move between inpatient and outpatient settings or try new medications (Marder et al., 2013). Negative symptoms that fluctuate to this degree are conceptualised as secondary negative symptoms in contrast to the more persistent and stable presentation that characterises primary negative symptoms (Mosolov & Yaltonskaya, 2022). No study reported the duration of participants' negative symptoms at enrolment. Without this information, it is not possible to understand if participants were experiencing transient secondary negative symptoms or persistent primary negative symptoms. Experts in the field have recommended that participants' negative symptoms are stable at the point of enrolment into research studies; 4 weeks of prospective stability has been suggested (Marder et al., 2013). Additionally, the gender imbalance throughout studies must be acknowledged as a potential confound. Males were overrepresented in clinical samples and underrepresented in non‐clinical samples.

### Conclusion

The present meta‐analysis has considered the relationship between attachment insecurity and psychosis‐spectrum negative symptoms. A small but significant positive association was found between attachment insecurity and negative symptoms in clinical samples. We also found evidence of a positive association between attachment insecurity and negative schizotypy in non‐clinical samples. This association was strongest between attachment avoidance and negative schizotypy. Considering each of the five symptoms individually revealed differing strengths of the relationship between attachment insecurity and negative schizotypy. For example, based on the three studies included in our review, anhedonia appears to have the strongest positive association with attachment avoidance. Whether these findings are related to conceptual overlap between measures is not clear, and any conclusions about these associations are limited by the methodological issues present in the synthesised studies. However, the consistency of effect sizes across studies utilising different measures of symptom‐specific NS and attachment dimensions indicates these experiences are associated.

Future research should prioritise the use of measures that adhere to the NIMH consensus definition, such as the SNS or CAINS, and should examine the relationship between attachment insecurity and individual domains of negative symptoms (Dollfus et al., 2016; Kirkpatrick et al., 2006). Until the literature is developed in this direction, the relationship between attachment and negative symptoms will remain obscured by methodological issues.

## AUTHOR CONTRIBUTIONS

**Aiden Duffy:** Conceptualization; methodology; data curation; writing – original draft; writing – review and editing; project administration; investigation. **David Carmichael:** Conceptualization; supervision; writing – review and editing. **Zsofia K. Takacs:** Formal analysis; writing – review and editing. **Regina Murphy:** Writing – review and editing; validation; data curation. **Sean Harper:** Writing – review and editing; conceptualization. **Tia McEwan:** Data curation; formal analysis. **Karen Goodall:** Conceptualization; writing – review and editing; supervision; validation.

## CONFLICT OF INTEREST STATEMENT

All authors declare no conflict of interest.

## Data Availability

The data that support the findings of this study are presented within the paper.
